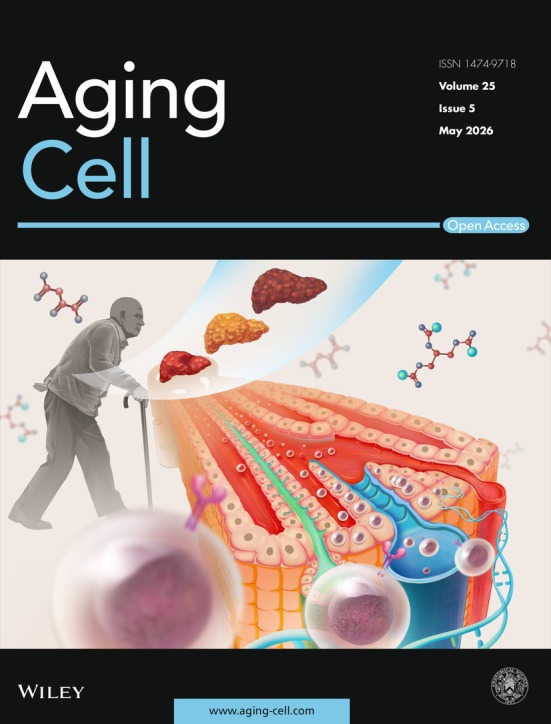# Featured Cover

**DOI:** 10.1111/acel.70532

**Published:** 2026-05-10

**Authors:** Jiahua Lu, Yuqian Wang, Wenxue Zhao, Zihao Zhao, Zhaoya Gao, Jin Gu, Cheng Li, Jie Cheng

## Abstract

Cover legend: The cover image is based on the article Spatiotemporal Transcriptomics Characterizes Immune Microenvironment During Mouse Liver Aging by Jiahua Lu et al., https://doi.org/10.1111/acel.70482.